# Spatiotemporal electrical dispersion mapping for substrate characterization and targeted ablation in patients with ventricular fibrillation

**DOI:** 10.1016/j.hrcr.2025.05.007

**Published:** 2025-05-13

**Authors:** Emanuel Heil, Nikolaos Dagres, Gerhard Hindricks, Jin-Hong Gerds-Li, Felix Hohendanner

**Affiliations:** 1 Department of Cardiology, Angiology and Intensive Care Medicine, Deutsches Herzzentrum der Charité (DHZC), Berlin, Germany; 2 DZHK (German Centre for Cardiovascular Research), partner site Berlin, Germany

**Keywords:** Ventricular fibrillation, Electroanatomic mapping, Dispersion mapping, Electrical storm, Ventricular substrate, Ablation

**Key Teaching Points**

• Artificial intelligence–supported spatiotemporal dispersion (stD) mapping effectively identifies arrhythmogenic substrates during active ventricular fibrillation (VF) in patients with hemodynamic support.
• Targeted ablation guided by stD mapping successfully prevented re-induction of VF, demonstrating procedural success and highlighting the method's potential clinical utility.
• StD mapping provides critical insights into substrate dynamics during unstable VF, overcoming limitations associated with traditional mapping techniques such as voltage and activation mapping.
• The clinical application of stD mapping during VF represents a novel approach that may enhance outcomes in complex ventricular arrhythmia ablation procedures.
• Integration of dynamic substrate assessments through AI-guided stD mapping can potentially improve ablation strategies, particularly in high-risk, unstable cardiac conditions.

## Introduction

Ventricular fibrillation (VF) is associated with high morbidity and mortality in patients with end-stage heart failure related to adequate internal cardioverter defibrillator (ICD) therapy or sudden cardiac death. It, therefore, remains a challenging arrhythmia characterized by disorganized electrical wavefront propagation and conduction disturbances. Mapping and ablation are complicated, leaving few non-pharmacologic options. Traditional mapping techniques, such as voltage and activation time mapping, which can be used particularly in patients with mechanical circulatory support devices such as left ventricular assist devices (LVADs), fall short because of the unstable and chaotic nature of VF. However, recent studies emphasize the critical role of electrical dispersion—variability in myocardial conduction velocity and repolarization—as a central mechanism underlying perpetuation of fibrillatory activity.^1^ Artificial intelligence (AI)–guided spatiotemporal dispersion (stD) mapping addresses these limitations by identifying dynamic electrophysiological heterogeneities reliably, fast, and automatically. Previous animal and preclinical studies have suggested that targeted modulation of dispersion may suppress arrhythmias. The technology has only recently demonstrate promise in a prospective study with patients with persistent atrial fibrillation (AF).^1^ However, clinical application during ongoing VF episodes has not been documented. This study explores the clinical translation of stD mapping during ventricular tachyarrhythmia ablation procedures, correlating dispersion findings with established electroanatomical metrics and clinical outcomes. The present observational study introduces a novel AI-supported stD mapping technique specifically applied during VF in patients with LVAD. The presented findings aim to demonstrate this mapping method's feasibility and efficacy in identifying arrhythmogenic substrates under the challenging and inherently unstable conditions of VF.

## Methods

We included 2 patients who underwent radiofrequency (RF) ablation at the German Heart Center of the Charité (DHZC) in Berlin, Germany, in 2024 in conjunction with stD mapping. Each patient underwent high-density, 3-dimensional (3D) electroanatomical voltage and activation (Pentaray, Carto, Johnson & Johnson, Irwindale), and stD mapping.^1^ The latter was performed using the Volta AF-Xplorer (Volta Medical, Marseille, France), a machine learning algorithm trained to detect local electrogram dispersion based on expert annotations. The software classifies each analyzed electrogram as either full dispersion (significant temporal variation in activation intervals and waveform, marked dark blue) or interim dispersion (marked white) on the 3D map. Regions labeled with full dispersion represent sites of substantial interbipolar spread of activation over the entire fibrillatory cycle length as annotated by the VOLTA system. Patients provided written informed consent, and the software was used within the local ethics committee approval EA2_145_24 framework.

**Figure 1:**
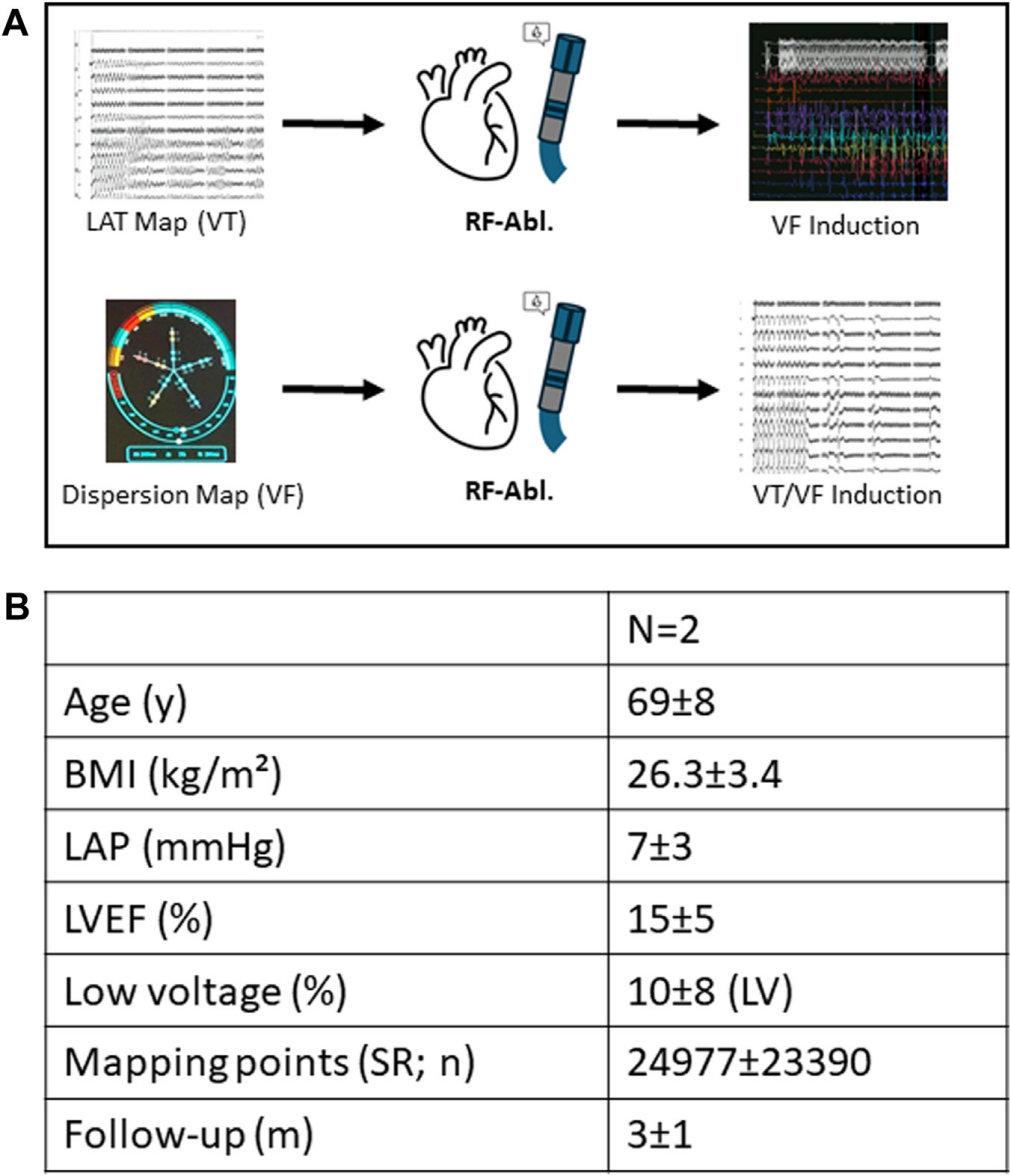
**A:** Protocol used for voltage, dispersion mapping, and ablation with RF-energy in patients with VF or electrical storm. **B:** Clinical characteristics of the patients. Abl = ablation; BMI = body mass index; LAP = left atrial pressure; LAT = local activation time; LV = left ventricle; LVEF = left ventricular ejection fraction; RF = radiofrequency; SR = sinus rhythm; VF = ventricular fibrillation; VT = ventricular tachycardia.

Initial electroanatomical voltage and activation mapping was performed during an atrial-driven rhythm (sinus rhythm or AF), followed by induction and mapping of ventricular tachycardia (VT). RF ablation was then carried out as indicated. This was followed by VF induction and subsequent stD mapping. During dispersion mapping, the VOLTA AF Explorer software annotated sites of full dispersion (marked dark blue) and interim dispersion (white). On the basis of these findings, additional dispersion-guided RF ablation (35 W, irrigated) was performed. All RF applications were delivered using an irrigated RF catheter (Thermocool SmartTouch SF; Johnson & Johnson) according to clinical routine. The procedural end point was non-inducibility of VT and VF, confirmed by reinduction testing (Figure 1A).

Primary demographic data included age, height, and weight. Left ventricular ejection fraction (LVEF) was measured by transthoracic echocardiography. Left atrial pressure was assessed on transseptal puncture.

## Case report

stD regions were identified in both patients with LVADs, complex ventricular tachyarrhythmias, and subsequent circulatory failure due to right heart dysfunction (Figure 1B).

The first patient, a 77-year-old woman, had a medical history significant for suspected myocarditis in the 1990s that progressed to dilated cardiomyopathy, secondary mitral and tricuspid regurgitation, and secondary pulmonary arterial hypertension. After negative coronary angiography result, a prophylactic ICD was implanted in 2011 because of severely reduced LVEF. The ICD delivered multiple appropriate shocks for ventricular tachyarrhythmias in the subsequent years. Pharmacologic rhythm management included amiodarone (discontinued due to side effects) and subsequently sotalol. In 2020, cardiac resynchronization therapy was implemented to address left bundle branch block, followed by a MitraClip placement in November 2022 for severe mitral valve regurgitation. Medical therapy was continuously optimized per guidelines.

By September 2023, because of further deterioration of systolic function (LVEF 10%–15%), a continuous-flow LVAD (Abbott HeartMate 3) was implanted. Histopathology of the resected ventricular apex during LVAD implantation confirmed dilated cardiomyopathy without active inflammation. In January 2024, the patient’s pacemaker documented an episode of VT (heart rate 250 bpm), which was refractory to antitachycardia pacing and required a 35-J shock. Despite an initial electrophysiological study and RF ablation targeting an LVAD inflow cannula-related substrate, the patient experienced further VT episodes (cycle length 230 ms) in October 2024, requiring a repeat ablation procedure.

The second procedure, conducted under deep sedation with near-infrared spectroscopy (NIRS) for cerebral oxygenation monitoring, used vascular access through the femoral vessels and a transseptal puncture. High-density electroanatomic mapping identified a basal septal substrate. Programmed electrical stimulation induced multiple unstable tachyarrhythmias, occasionally degenerating into VF, with no clear targets from standard voltage mapping. Furthermore, stD mapping during induced VF identified a dispersion-positive zone near the left anterior fascicle on the septum, corresponding to subtle low-voltage signals in sinus rhythm (bipolar voltage in stD regions: 2.0 ± 0.4 mV). As NIRS-reported cerebral oxygenation decreased significantly during the course of 6 minutes of VF mapping, the patient was defibrillated. Subsequent targeted RF ablation at the identified site successfully prevented reinduction of sustained arrhythmia using a ramp pacing protocol (cycle length 300–150 ms).

**Figure 2:**
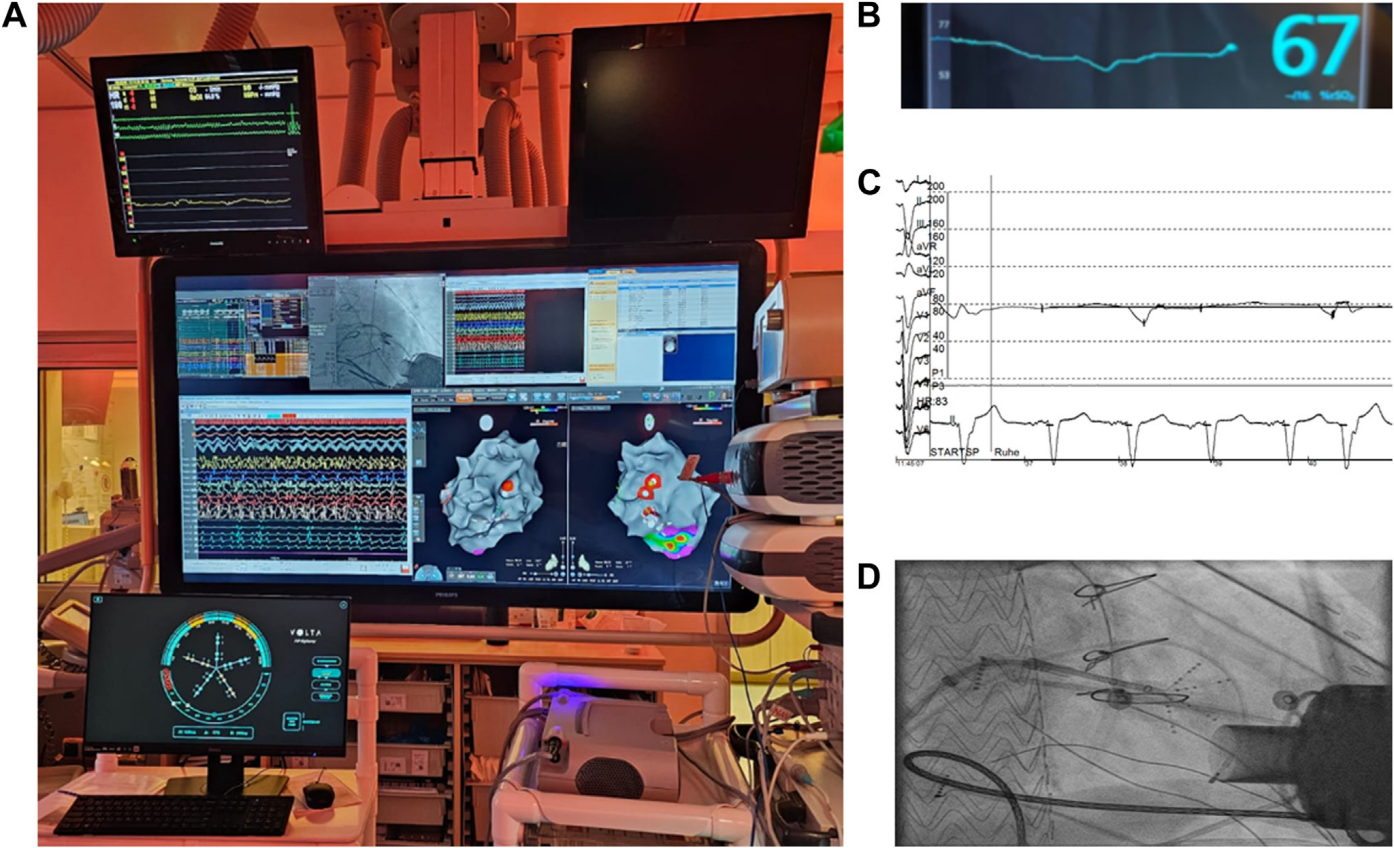
**A:** EP laboratory setup with **B:** NIRS and **C:** hemodynamic monitoring. **D:** AP projection during mapping (radiograph). AP = anteroposterior; EP = electrophysiology; NIRS = near-infrared spectroscopy.

The second patient, a 71-year-old man with end-stage ischemic cardiomyopathy, underwent LVAD implantation after recurrent VF and ICD implantation post-electrical storm. Subsequent device explantation was necessitated by suspected infection and persistent bacteremia, with a negative microbiological evaluation result despite a pump-area abscess. Due to persistent hemodynamically significant VF episodes, another electrophysiological study was performed under sedation with NIRS monitoring (Figure 2B). After exclusion of progressive ischemic disease by coronary angiography, mapping identified an inferior low-voltage substrate near the LVAD cannula, exhibiting significant late potentials. Induced monomorphic VT (cycle length 210 ms) displayed a superior axis and negative concordance. Activation mapping localized a critical isthmus from mid-inferior to apical-inferior regions within an aneurysmatic segment. VF was induced through programmed stimulation, followed by stD mapping. This revealed significant dispersion at the apical border (bipolar voltage amplitude 1.2 ± 0.3 mV), where mid-diastolic potentials were also detected. Ablation with an irrigated RF catheter modified the substrate, prolonging VT cycle length to 250 ms, though the arrhythmia subsequently destabilized into VF. Additional dispersion mapping during VF identified altered electrical properties near the cannula in the anterolateral area, guiding further ablation. Subsequently, extensive RF applications targeted dispersion-positive regions without terminating VF. Sinus rhythm was restored using electrical defibrillation, and a ventricular ramp pacing protocol (cycle length 250–200 ms), with no further induction of VT or VF, indicated procedural success.

**Figure 3:**
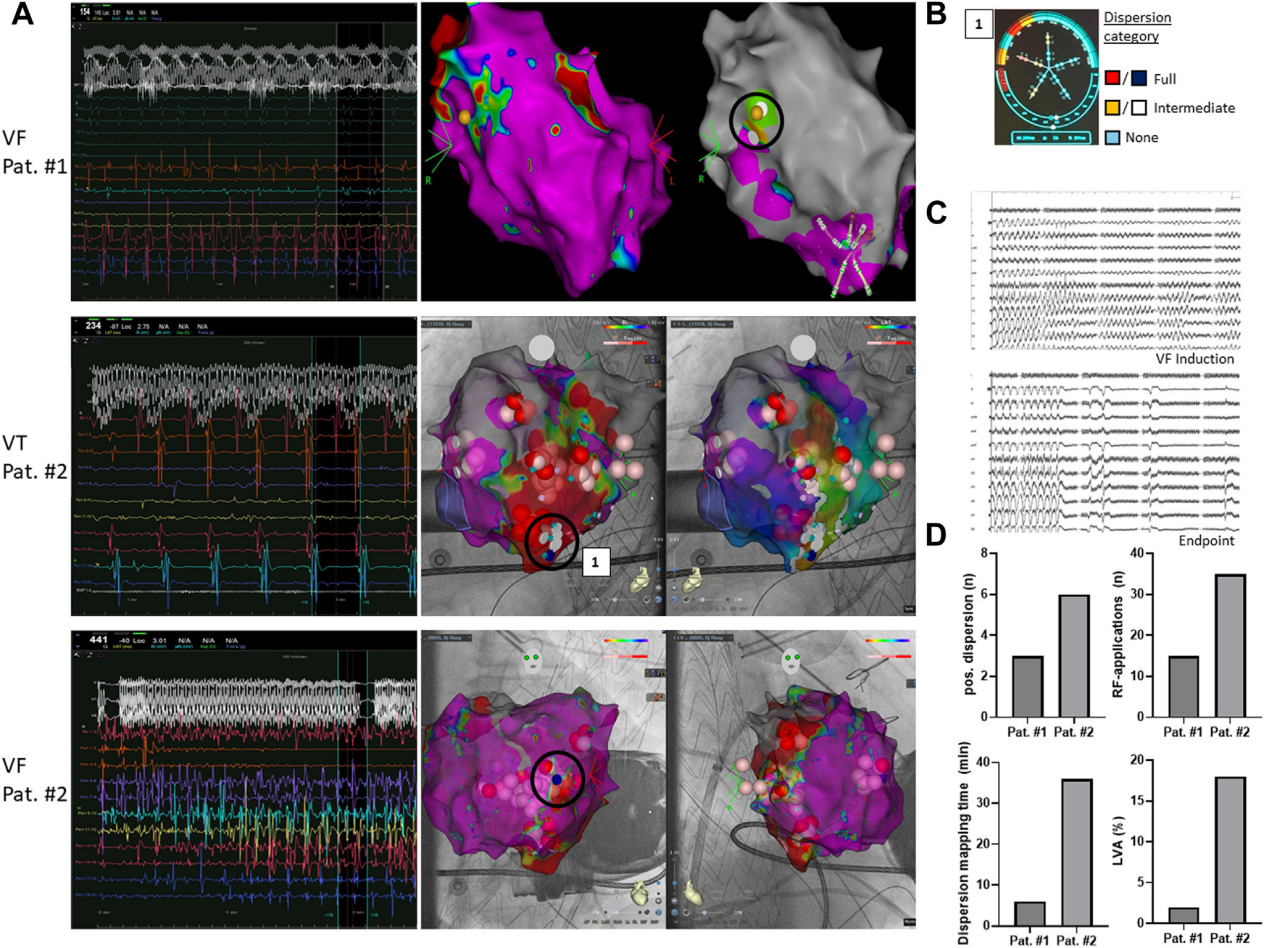
Spatiotemporal dispersion mapping of ventricular tachyarrhythmia. **A:** Top. VF map of patient #1 and the respective 3D electroanatomic map (*left:* SR, *right:* VF) with dispersion positive signals (*black circle*; corresponding electrical signal: *left*) in close proximity to a low voltage region. **A:** Center. VT map of patient #2 with a dispersion positive region (1/*black circle/white and blue dots*) in close proximity to a LAT mapping delineated critical isthmus (right). **A:** Bottom. VF map with dispersion positive region (*center/blue dot*) and the respective electrical signals (left). Shaded *red and pink dots* mark RF applications. **B:** Dispersion analysis tool with positive signals at the respective site (1). **C:** VF induction protocol and end point of the procedures. **D:** Quantification of procedural characteristics. RF = radiofrequency; SR = sinus rhythm; VF = ventricular fibrillation; VT = ventricular tachycardia.

Overall, 9 dispersion-positive regions were identified in VF, guiding 50 RF ablation applications. Dispersion mapping times during VF were 6 minutes for patient one and 36 minutes for patient two, with overall left ventricular low-voltage areas of 2% and 18%, respectively (Figure 3). No further episodes of VT or VF occurred during a mean follow-up of 4.5 ± 0.5 months. Electrogram recordings on the multipolar catheter placed at dispersion-positive regions during VF and in SR are found in Supplemental Figure 1. Detailed electrograms of dispersion-positive regions and the respective sets of RF ablations targeting either dispersion region (VF map) or the critical VT isthmus are highlighted in Supplemental Figure 2.

## Discussion

This observational study underscores the promising clinical utility of stD mapping in VF, especially among patients supported by LVADs. To our knowledge, this represents the first clinical report of stD mapping applied successfully for guiding substrate-based ablation during active VF episodes, demonstrating procedural success through targeted ablation of dispersion-positive regions. It also supports the dogma of intracardiac electrogram clustering as a driver of VF in heart failure.

Previous evidence has established stD as a valuable marker for advanced or heterogeneous substrate remodeling in AF. Specifically, the TAILORED AF trial demonstrated that ablation of stD-positive regions significantly reduced AF recurrence rates after a single procedure.^1^ However, VF presents more intricate pathophysiological mechanisms than AF, involving local automaticity, wavefront fragmentation, re-entry circuits, and conduction blocks—all driven by heterogeneous myocardial conduction and repolarization.^2^, ^3^, ^4^, ^5^

Traditional high-density electroanatomical mapping is frequently constrained by VF's inherently chaotic dynamics and rapid hemodynamic instability. Furthermore, manual mapping of dispersion, particularly within atrial contexts, is inherently subjective and highly dependent on operator experience.^6^ AI-supported stD mapping mitigates these challenges by providing automated, dynamic identification of electrophysiological dispersion, thus facilitating more precise localization of critical arrhythmogenic substrates.^7^

Existing literature emphasizes the critical role of targeted ablation of VT isthmuses. However, in more complex ventricular tachyarrhythmias, including VF, these traditional isthmuses identified through voltage mapping, activation sequences, and pace-mapping maneuvers often do not serve as critical maintenance sites. The incremental value of stD mapping lies in its capacity to identify functional substrates of electrophysiological significance that traditional techniques might overlook. These dispersion-positive areas may not necessarily correspond to conventional critical isthmuses, as their significance may emerge from the broader substrate context. Our findings reinforce this concept by demonstrating procedural success, characterized by VF non-inducibility, in 2 patients after targeted ablation of stD-positive regions. However, termination of VF itself does not seem to be a feasible clinical end point in this set of patients. This is potentially related to the fact that dispersion might reflect the presence of drivers that are more relevant for VF initiation than maintenance.

Moreover, preclinical studies strongly support the significance of electrical dispersion in initiating and sustaining VF, with pharmacologic strategies primarily targeting dispersion reduction to prevent VF episodes.^8^^,^^9^ Therefore, the preliminary clinical results presented here align well with preclinical data, suggesting the clinical potential of dispersion-targeted therapeutic approaches. For interventional mapping, hemodynamic support using LVADs such as Impella or HeartMate, or circulatory support systems such as ECMO, is essential to maintain stability. In our cases, NIRS proved valuable in assessing the patient's central oxygenation during the procedure.

Despite these encouraging observational results, larger-scale studies are essential to confirm the reliability, generalizability, and effectiveness of stD mapping in broader patient populations with complex ventricular tachyarrhythmias. Future research should focus particularly on validating these findings across diverse clinical scenarios and establishing standardized protocols for integrating dispersion mapping into routine clinical practice.

## Conclusions

Our initial experience highlights that stD mapping provides a clinically relevant tool for delineating arrhythmogenic substrates during active VF. By integrating dynamic substrate assessments into ablation strategies, stD mapping has the potential to significantly enhance procedural outcomes and improve clinical management in high-risk VF patients.

## Declaration of Generative AI and AI-Assisted Technologies in the Writing Process

During the preparation of this work, the authors used ChatGPT 4.5 to improve language. After using this tool/service, the authors reviewed and edited the content as needed and take full responsibility for the publication's content.

## Disclosures

The authors have no conflicts of interest to disclose.
