# Supplementary material for: Spatiotemporal electrical dispersion mapping for substrate characterization and targeted ablation in patients with ventricular fibrillation
Source: HeartRhythm Case Rep. 2025 May 13;11(8):717–21. doi: 10.1016/j.hrcr.2025.05.007 (PMC12399165; doi:10.1016/j.hrcr.2025.05.007)
Supplement: Supplementary Figures 1 and 2 [file mmc1.docx]

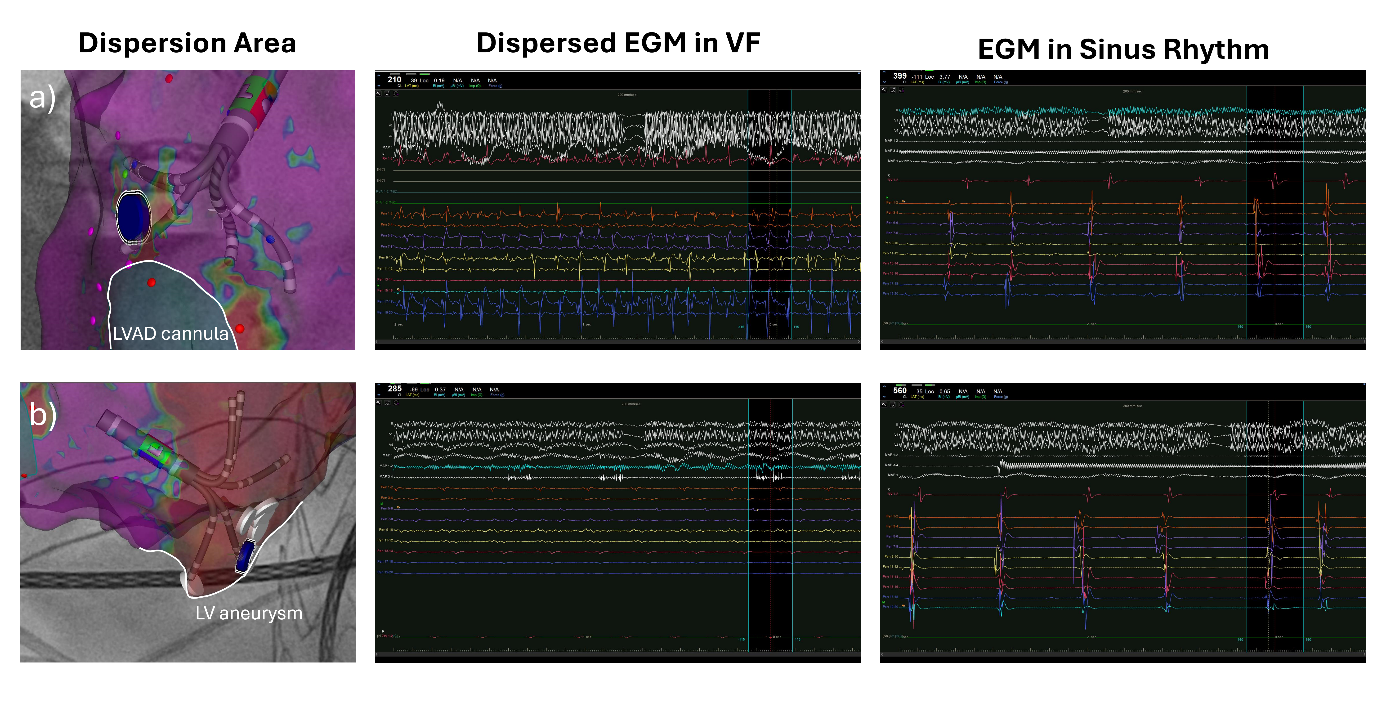


Suppl. Figure 1: EGM recordings on the multipolar catheter placed at dispersion positive regions 1 (a) and 2 (b) during VF and in SR (patient #2).


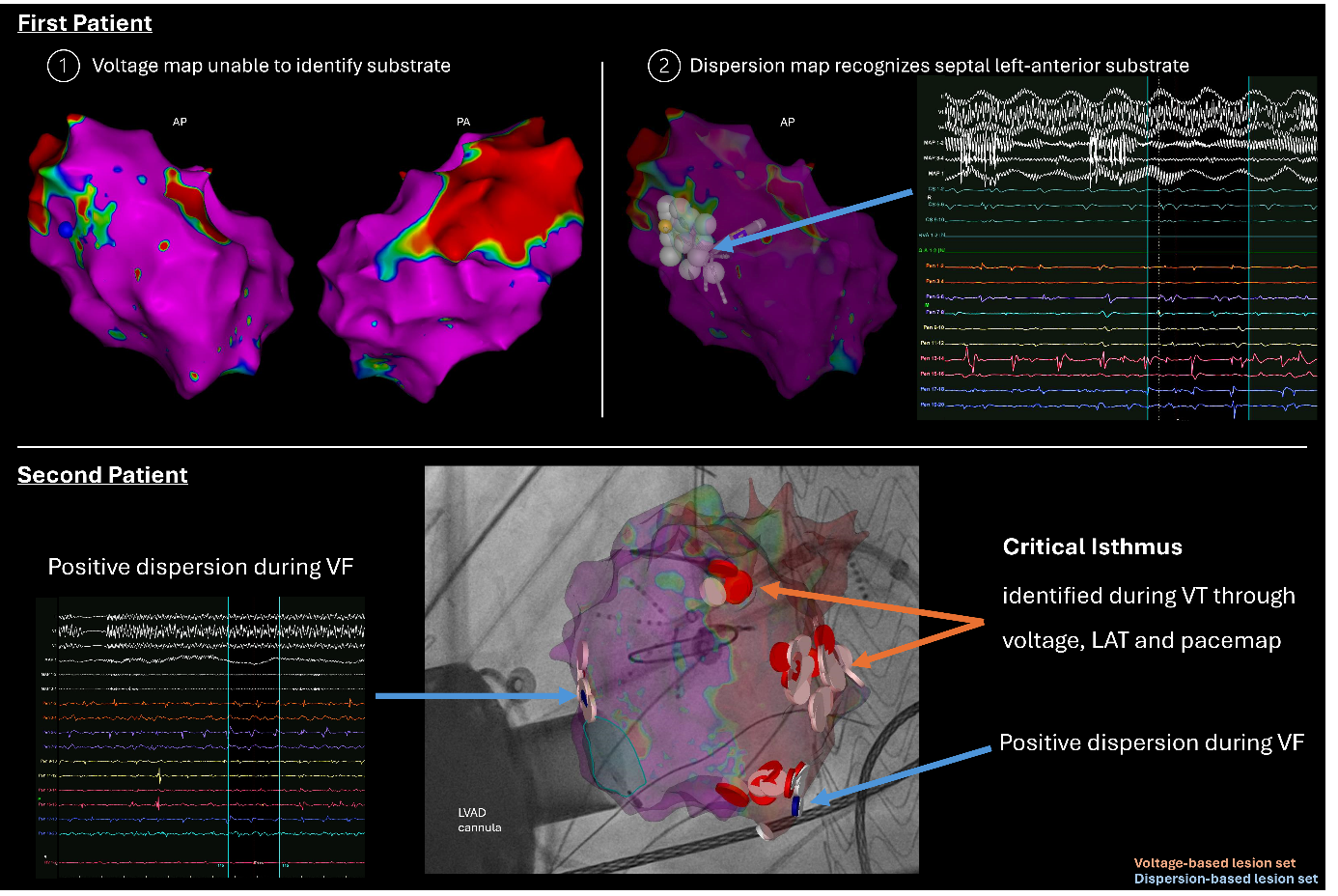


Suppl. Figure 2: Dispersion positive regions in patient #1 (top) and #2 (bottom) and the respective sets of RF ablations targeting either dispersion regions (VF map) or the critical isthmus of a VT (LAT/Voltage map).
